# Tele-Physical Activity Promotion Program among College Students during the COVID-19 Pandemic

**DOI:** 10.3390/medicina59020332

**Published:** 2023-02-10

**Authors:** Reem M. Basuodan, Bodor H. Bin sheeha, Nada E. Basoudan, Nada A. Abdljabbarl, Monira I. Aldhahi

**Affiliations:** 1 Department of Rehabilitation Sciences, College of Health and Rehabilitation Sciences, Princess Nourah bint Abdulrahman University, P.O. Box 84428, Riyadh 11671, Saudi Arabia; 2 College of Health and Rehabilitation Sciences, Princess Nourah bint Abdulrahman University, P.O. Box 84428, Riyadh 11671, Saudi Arabia

**Keywords:** health promotion, pandemic, sport, Saudi Arabia, physical activity

## Abstract

*Background and Objectives:* During the COVID-19 pandemic, lockdown and distance learning affected physical activity (PA) levels among college students. The aims were to assess the effectiveness of a tele-health PA promotion program for 6 weeks, among junior college students, on PA level and on the proportion of physically active students during the pandemic. *Materials and Methods:* A pre–post study design was conducted on 46 students aged 19 (±0.9) years old in Saudi Arabia. The study consisted of online introductory and educational PA classes, followed by a 6-week course during which students received daily online PA promotive messages. Wilcoxon signed-rank and McNemar’s tests were used to measure the mean differences in PA level and the changes in proportion of physically active students before and after the program, respectively. *Results:* The proportion of students who perform walking increased significantly from 47.4% to 68.4% (*p* = 0.02), while the number of students who perform moderate PA in their leisure time increased significantly from 38.9% to 69.4% (*p* = 0.02). No significant differences were detected between other PA levels. *Conclusions:* This program is effective in encouraging more college students to be physically active, but not in improving PA levels. Larger scale studies using PA objective measurement tools are needed.

## 1. Introduction

Physical activity (PA) is essential to maintain health and to decrease the risk of many non-communicable diseases, such as cardiovascular disease, hypertension, thromboembolic stroke, diabetes mellitus, osteoporosis, colon cancer, and breast cancer [1]. Therefore, global health agencies make tremendous efforts to promote PA to reduce sedentary behavior, improve wellbeing and quality of life, and prevent disease [2]. Junior college students face considerable challenges during the transition to study life, particularly in maintaining a healthy lifestyle due to stress and a lack of time, leading to a decrease in PA levels and performance compared to their levels and performance during secondary school [3,4].

In Saudi Arabia, the proportion of non-physically active females was recorded as 78%, which is remarkably high [5]. It has been reported that the proportion of overweight students increased from 21% at the beginning of the first year of college to 32% at the end of that year, in the United States [6]. Overweight and obesity during adolescence quadruples the risk of being obese as an adult in comparison to normal weight adolescents [7]. This emphasizes the need to develop healthy campuses and PA interventions in the context of college life to motivate students to increase their PA level. Interestingly, PA patterns while at college influence the physical behavior of adults and are maintained for a long time afterward [8,9]. Therefore, a healthy campus can help students and communities to adopt active lifestyle behaviors. The efficiency of a PA intervention program among college students was assessed in a systematic review and meta-analysis, where the evidence shows significant PA improvement following different forms of interventions [10]. In Saudi Arabia, there is a consensus on the urgent need for a PA promotion program for college students, as their PA levels are below the recommended, with high percentages of overweight and obesity [11,12,13,14].

Restrictions during the COVID-19 pandemic were implemented to mitigate the spread of the virus worldwide, which led to a significant decrease in exercise participation and an increase in sedentary time among the public, including college students, despite the positive effects of exercise during the pandemic restrictions [15], such as in boosting the immune system [16] and reducing the risk of developing severe COVID-19 symptoms [17]. In Saudi Arabia, Riyadh had the highest percentage of new daily cases of COVID-19 (average 24.2%) [18], and strict measures such as physical distancing and home stay were implemented in March 2020 [19]. A tele-health PA intervention program was proposed during the pandemic to maintain safety recommendations and reduce transmission of the virus [20]. In addition, the evidence shows that using tele-health promotion programs is effective for health behavior modification [21,22,23,24,25]. Usually, the tele-health PA promotion program is delivered using advanced applications for smartphone users or costly smartwatches, which may be unfeasible for all users due to age or financial level [26]. The novelty of current study is assessing the effectiveness of a tele-health PA promotion program for 6 weeks on PA level among junior college students, and on the proportion of active students during the COVID-19 pandemic using free smartphone applications.

## 2. Materials and Methods

### 2.1. Study Design and Participants

**Figure 1:**
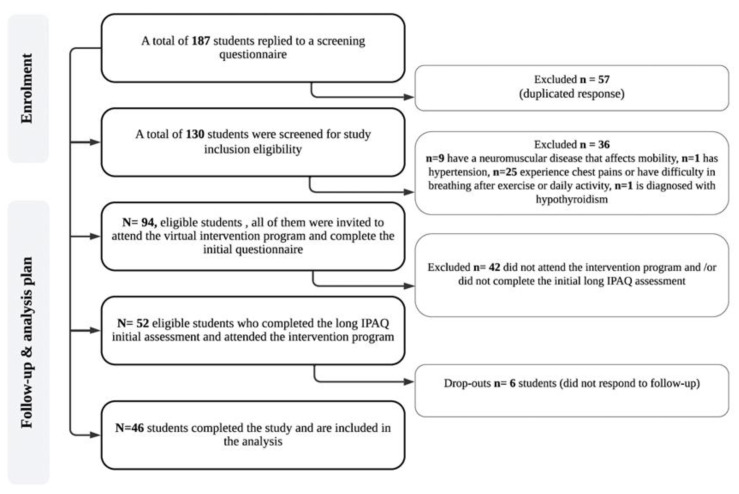
Study flow diagram.

A pre–post study design was conducted at the College of Health and Rehabilitation Science at Princess Nourah bint Abdulrahman University in Saudi Arabia. Junior female college students (n = 187) were recruited via convenience sampling during the fall 2020 semester. Data were collected from September, which is the beginning of the academic year, to November 2020. Students included in the study were studying remotely due to the lockdown measures that were implemented in Riyadh due to the COVID-19 pandemic. The inclusion criteria were healthy junior college students aged from 18 to 21 years old. All participants were assessed for health conditions through a medical screening survey. For student screening, we conducted an anthropometric assessment and medical screening. The anthropometric assessment included weight in kilograms, and height in centimeters. The medical screening included a question about the presence of medical history with six health problems provided as answers; these were the following: cardiovascular, neurological, orthopedic, diabetes, and other health problems. It also included closed questions about pain during movement, musculoskeletal injury, high blood pressure, and current medications, if any. The screening questions were self-recorded by each participant using an online format. All students needed to participate in all the study phases and to have completed all assessments to be included in the data analysis. Of the 187 recruited students who completed the screening survey, 94 were eligible but only 46 completed the study requirements (Figure 1). The mean (±SD) age and BMI of the students were 19 (±0.9) years and 21.9 (±4.44) kg/m^2^, respectively.

This study was conducted in accordance with the guidelines proposed by the Declaration of Helsinki and was approved by the Ethics Committee of Princess Nourah bint Abdulrahman University, Riyadh, Kingdom of Saudi Arabia (No 20-0301). All methods were carried out in accordance with relevant institutional review boards and regulations. The study protocol, procedures, and respondents’ rights were explained at the beginning of the study. Informed consent was obtained from all respondents prior to completing the survey.

### 2.2. Procedure

#### 2.2.1. Tele-Physical Activity Program

The study comprises two phases: an educational phase and an interventional phase. Students who agreed to participate in the study and met the inclusion criteria were included in the educational phase, which involved a virtual introductory PA class via an educational platform for an hour. In the introductory session, an induction class was conducted in which the students were instructed about the program and the physical activity guidelines. This included knowledge about physical literacy to help the students to cultivate a commitment to lifelong healthy, active living. In addition, the physical and mental benefits of physical activity were highlighted.

Before starting the tele-physical promotion program, a test run was performed on the ‘WhatsApp.’ application to ensure that the suitability and the promotive card deployment were optimal. Each student was instructed on how to download and setup the application. The intervention program comprised 5 sessions per week over 6 weeks for a total of 30 sessions. During the session, students received an online promotive message via a smartphone application ‘WhatsApp.’ Promotive messages were designed to incorporate the fundamental principles of physical activity, benefits of physical activity on academic achievement, mode of volitional/nonvolitional physical activity, work in a variety of group contexts, and the availability of appropriate material and equipment used at university, motivating them to engage in regular PA. The promotional messages were displayed on the card as illustrations and written content.

Physical activity promotion messages were created by a certified physical therapist panel, and were unified for all students and sent at the same time on daily basis. These also provided recommendations and plans for the PA structure and self-managed exercises. One message was deployed every working day (5 messages/week). These patterns were found to maintain adherence according to McAuley [27]. Both phases were prepared and provided by the Department of Rehabilitation faculty at Princess Nourah bint Abdulrahman University.

#### 2.2.2. Physical Activity Instrument

To measure the PA levels, two forms of international physical activity questionnaire (IPAQ) were used; IPAQ-long form (LF) and IPAQ-short form (SF). Both are self-administered 7-day recall questionnaires in the Arabic language and are intended to indicate population health-related PA. PAQ-LF includes five domains: job-related physical activity; active transportation physical activity; housework, house maintenance, and caring for family; recreation, sport, and leisure-time physical activity; and sitting. The IPAQ is commonly administered internationally for therapeutic and research purposes. Following extensive reliability and validity testing across 12 countries, the test has been shown to have an acceptable measurement of repeatable data for the short questionnaire version (Cronbach Alpha  = 0.80) [28]. In addition, the concurrent validity for the IPAQ-SF showed reasonable agreement when compared to the IPAQ-LF (Spearman’s correlation coefficients (rs) = 0.76) [29]. The IPAQ-SF allows for the calculation of metabolic costs in metabolic equivalent units (METs) [30]. The operational calculation of the metabolic equivalent in minute per week was performed by multiplying the MET value, which was based on a compendium of an average MET score for each type of activity (walking = 3.3, moderate activity = 4, vigorous activity = 8) [30] by the minutes the activity was carried out, and by the number of days on which that activity was undertaken. The formula for Computation of MET-minutes = MET value ×duration of activity (in minutes) × frequency per week. The changes in the proportion of physical activity were assessed in this study. Physical inactivity refers to non-achievement of the physical activity guideline, while physical activity refers to students performing PA at least one day per week for more than 30 min for each pattern.

### 2.3. Data Analysis

Data cleaning and participant stratification were conducted in accordance with the data processing guideline of the IPAQ. The collected data were screened to identify missing data, outliers, and normality. Data were considered missing if the participants left an item blank or marked the “I don’t know or refuse” answer. A pairwise deletion technique was used to treat missing data. In the data cleaning process, 48 students in total were excluded, as their data were missing (Figure 1). The Shapiro–Wilk test was performed to test the normality assumption of the data. The normality assumption is violated for all questions related to walking, vigorous and moderate MET; as a result, nonparametric testing was conducted. The main outcome data were collected at baseline and post-program immediately. Data are presented as medians (MED) and interquartile range (IQR) for continuous variables, and frequency and percentage (%) for categorical variables. The McNemar’s test was used to assess the changes in PA proportion of participants before and post-program. The Wilcoxon signed-rank test was used to assess whether there was a difference in PA before and post-program. Statistical significance was set at *p* ≤ 0.05. The effect size was calculated based on the point biserial correlation coefficient test. Collected data were analyzed using JMP software (JMP^®^, Version 15 SAS Institute Inc., Cary, NC, USA).

## 3. Results

The 6-week tele-physical activity promotion program showed a significant increase in the proportion of students who perform walking for transportation, and who perform moderate PA in their leisure time. The promotion program showed improvement in IPAQ-LF in the transportation, housework, recreation, and sports domains, and moderate activity in the IPAQ-SF. However, they did not reach a statistically significant level.

**Figure 2:**
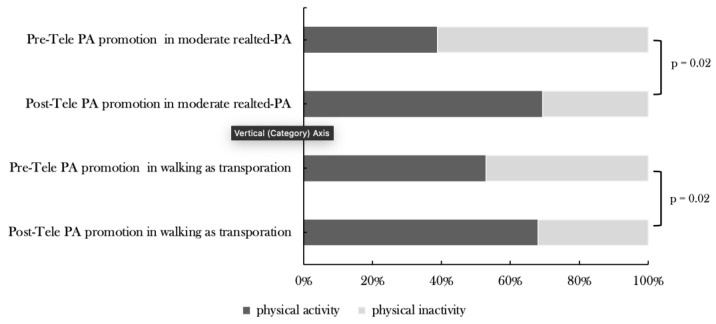
Proportional of changes in the number of students engaged in physical activity and physical inactivity following promotional program. PA = Physical activity.

In Figure 2, the McNemar’s test determines the proportion of students who are involved in PA following the 6-week tele-physical activity promotion program. Significant improvement has been reported in two domains: walking for transportation and moderate PA for leisure, with all *p*-values = 0.02. However, the proportion of students who are involved in PA in the remaining PA domains did not show significant change. As Figure 2 shows, there is a clear trend of increase in the proportion of students (47.4% (18 students)) participating in walking as transportation. Following the promotion program, the percentage of those students increased significantly to 68.4% (26 students). Similarly, the proportion of changes in students participating in moderate PA in their leisure time improved significantly from 38.9% (14 students) pre-telePA promotion program to 69.4% (25 students) post-telePA promotion program (Figure 2).

The descriptive statistics for the IPAQ-LF relating to vigorous and moderate activity, walking, and the metabolic equivalents (METs) for each domain pre and post-program are presented in Table 1. For the IPAQ-LF findings, the Wilcoxon signed-rank test elicited no significant difference between all PA levels, and METs in all domains (Table 2). In the active transportation domain, the reduction in the time spent in vehicles and the increase in the time spent walking or bicycling (particularly for transport) was apparent; yet, there was lack of statistically significant findings. In the housework domain, the non-significant increase in the days spent doing moderate PA in the garden and at home led to an improvement in the median moderate PA, and subsequently the MET of 22 min/wk and 141, respectively. In the recreation and sports domains, the non-significant increase in the days and time spent walking and in moderate and vigorous PA as leisure activities led to an improvement in the median moderate PA, walking, and the MET of 25 min/wk, 30 min/wk, and 77 min/wk, respectively (Table 1).

The IPAQ-SF findings showed no significant changes between the two assessments in terms of vigorous, moderate, walking, and sitting times. However, the median time of moderate activity increased by 15 min/week for moderate PA and by 30 min/week for walking. There were no changes in vigorous activity and sitting after the promotion program (Table 3).

## 4. Discussion

The current study showed that a six-week tele-physical activity promotion program was effective in increasing the proportion of students who perform walking for transportation, and those who perform moderate PA in their leisure time, but PA levels detected by IPAQ-LF showed no significant improvement. According to a systematic review conducted before the pandemic in 2018, it has been recorded that there are considerable percentages of inactivity among college students in Saudi Arabia [14]. Prior studies have reported physical inactivity in Saudi Arabia [12,13]. Many studies shed light on the negative impact of the COVID-19 pandemic on PA levels and sedentary behaviors in both genders at all ages, globally and in Saudi Arabia [15,31,32,33]. Our study was conducted just after partial release from the complete lockdown, where restricted access to many facilities, including sports venues and recreation centers, was applied. In addition, distance learning and many other restrictions were still applied as well. McCarthy et al. [34] concluded that PA level recovery after COVID-19 restrictions were removed was not a spontaneous processes. They believe that more effort is needed to return to normal routine and the PA baseline [34]. In our study, we started up with an online introductory class followed by educational class via an online platform; then, we sent daily smartphone messages about PA via WhatsApp for 6 weeks. We found that the number of students who performed moderate PA in their leisure time increased significantly after the program.

Our study showed that sending messages via smartphone WhatsApp increased students’ proportions who performed moderate PA using smartphones. Previous tele-health research found that mobile intervention programs, such as through WhatsApp, are useful and accepted among Saudi college students and are an effective tool to be used in PA level improvement in female participants [21,22,23]. One possible reason behind this improvement is that our tele-physical activity promotion program utilizes WhatsApp, which is widely used in Saudi Arabia, to send the educational messages on a daily basis [13]. Also, the usage of an electronic health intervention method showed to be a better alternative during the period of the pandemic than the previously available options for PA activities [25]. Knowledge about PA in our study was provided in the form of online messages and a virtual class which were believed to be effective educational tools to produce behavioral change in PA among students [35]. In addition, Hillsdon et al. [36] concluded that attending PA classes in facilities is not necessary to obtain sustainable increases in PA levels. Furthermore, PA interventions based on online rather than face-to-face interventions positively affect walking activities [37,38]. In our study, the nature of the intervention is online, which might overcome some PA barriers, such as the time required for face-to-face interventions [39]. Messages that have been sent in our study focused on enhancing general PA knowledge among college students in order to improve their PA levels, which is one method of PA promotion that has been implemented in previous studies [40,41]. Studies showed that participants became more motivated when they knew more about PA health benefits, which could increase their perceived autonomy for PA participation [40,42]. Chen et al. [41] concluded that encouraging youth to move is associated with educating them about PA. In addition, in accordance with our study, in terms of sending daily PA messages, Peyman et al. found that the method of sending daily PA messages to participants influenced the PA behavior positively [21].

Our study did not show any significant improvement in the self-reported walking duration and PA levels. Globally, there has been a decrease in worldwide walking and daily steps because of the COVID-19 pandemic, during which young people stayed at home and had less walking activity, as well as older people and especially women. [43,44]. It is not surprising that during COVID-19, the daily routine activities including PA might be affected due to outdoor inaccessibility. Studies showed that an outdoor environment is important to motivate and facilitate PA [45]. A recent study conducted in Saudi Arabia showed that doing PA alone or in groups with a personal trainer during the COVID-19 pandemic can prevent the drop in PA level in terms of MET min/wk of PA before and during the lockdown [46]. However, the tele-physical activity program in this study has not included a personal trainer nor instructions about performing PA in groups. On the contrary to our finding, a 3-week mobile phone-based intervention directed at sedentary women increased their number of steps and their PA level [47]. Although part of their intervention was aligned with the promotion program used in this study, in which motivational messages were sent daily to students’ phones, their study was conducted before the COVID-19 pandemic and their different target age might contribute to the different results. A national survey for PA and sedentary behaviors, conducted during the COVID-19 restraint period, showed a significant decrease in active transportation [31] which, interestingly, in our study was found to be improved in terms of the proportion of students who perform walking for transportation compared to before the intervention. This could be due to the fact that the university campus offers transportation options between college buildings including the metro, private cars, or walking. Students in this study reported that they walk more for transportation after our PA promotional program.

The findings of the present study have some limitations. First, the questionnaire is a self-reported questionnaire which does not provide an insight on the direction of changes and the physiological mechanisms that contribute to these changes. Additionally, the nature of the self-reported questionnaires may introduce inevitable recall and social desirability bias. A self-perceived PA questionnaire carries some limitations as it does not consider the relative intensity of individual PA, interindividual and intraindividual variations such as sex, age, body mass, and level of fatigue. Additionally, some items of the long version of the questionnaire related to job physical activity, housework, house maintenance, and caring for the family might be influenced by the student’s age and impose threats to the external validity. Furthermore, the instrument used was not designed to describe the categories of volitional activities (e.g., planned, structured exercise), and nonvolitional activities (e.g., the, daily living activities). Further study is required to use standardized instruments (e.g., motion sensors, accelerometers, heart rate recorders, or oxygen consumption meters) to counteract the existing bias. Second, our intervention program did not attempt to provide direct supervision to the students to check the adherence to the intervention, which should be considered in prospective studies. Third, this is a single-center study with a small sample size and with no control group; thus, the results drawn from this population may not be generalizable to all university students. Therefore, the use of a diverse and larger sample and a randomized controlled research design are recommended for future studies. A future study designed for a longer duration is also recommended. Finally, we recommend a future study that includes both male and female students to explore the efficiency of the intervention for both genders. Including both genders may clarify the efficiency of the program and take into consideration the hormonal factors that may affect physical performance, such as hormonal cycle fluctuation and differences in body and muscle mass.

## 5. Conclusions

This study showed that a six-week tele-physical activity promotion program using smartphone WhatsApp messages to send PA information on a daily basis to college students during the COVID-19 pandemic did not improve their PA levels but elevated the percentage of students who perform moderate PA and walk for transportation. Since this program has no effect on students’ PA levels, more tele-PA promotion studies on larger scales and for longer program periods are needed to confirm our results.

## Figures and Tables

**Table 1:** International Physical Activity Questionnaire (IPQA-LF) changes following the Tele-Physical Activity promotion program.

IPAQ Domain		Pre-Promotion Program		Range	Post-Promotion Program		Range	*p*-Value	Effect Size
		MED	IQR		MED	IQR			
Job-Related Physical Activity *	Vigorous activity (min/wk)	0	0	60	0	0	0	0.32	
	Moderate activity (min/wk)	0	0	600	0	0	30	0.55	
	Walking(min/wk)	0	0	1440	0	0	360	0.57	
	METs	0	0	4266	0	0	1308	0.57	0.07
Transportation Physical Activity	Vigorous activity (min/wk)	-	-	-	-	-	-		
	Moderate activity (min/wk)	0	0	0	0	0	30	0.08	
	Walking (min/wk)	15	120	840	60	150	420	0.30	
	METs	49.5	396	2772	198	495	1566	0.34	0.03
Housework, House Maintenance, And Caring for Family	Vigorous activity (min/wk)	-	-	-	-	-	-	-	
	Moderate activity (min/wk)	60	360	1560	82.5	252.5	780	0.65	
	Walking (min/wk)	-	-	-	-	-	-	-	-
	METs	150	1338.75	5700	291	1026.8	3240	0.81	0.1
Recreation, Sport, and Leisure-Time Physical Activity	Vigorous activity (min/wk)	0	0	300	0	30	360	0.23	
	Moderate activity (min/wk)	0	35.25	245	25	69.75	840	0.14	
	Walking (min/wk)	30	165	700	60	180	420	0.08	
	METs	252	796	2994	329	892.5	4746	0.09	−0.08

Abbreviation: min/wk = minutes/week, MED = Median, IQR = Interquartile range, METs = Metabolic equivalents. * Most of the responses (95.7%) to the job-related PA domain (questions 1–7) were answered “No” except for two students. - No scoring for this intensity based on IPAQ scoring protocol. No Vigorous and moderate activity for sitting domain.

**Table 2:** Comparison of changes in the physical activity METs pre and post-Tele-Physical Activity promotion program.

Type of PA ^a^	Phase	METs			*p*-Value
		(MED)	IQR	Range	
Vigorous	Pre	0	0	2400	0.43
	Post	0	240	2880	
Moderate	Pre	180	1545	8991	0.88
	Post	512	1040	5190	
Walking	Pre	321.75	1188	2772	0.38
	Post	594	825	3168	

Abbreviation: MED = Median, MET = Metabolic Equivalents a. denotes using long International Physical Activity Questionnaire (IPQA-LF).

**Table 3:** Differences between the interim assessment during Tele-Physical Activity promotion program.

Type of PA ^a^	Assessment of IPAQ-SF	Time (MED)	METs (MED)	METs IQR	Range	*p*-Value	Effect Size
Vigorous	2nd week	15 min/wk *	120	480	2400	0.36	0.04
	4th week	15 min/wk	120	480	2880		
Moderate	2nd week	45 min/wk	180	315	960	0.79	−0.16
	4th week	60 min/wk	240	420	2520		
Walking	2nd week	120 min/wk	396	594	4158	0.85	0.01
	4th week	150 min/wk	* 495	7755	2706		
Sitting	2nd week	7 h/day	**	**	**	0.45	−0.18
	4th week	7 h/day					

Abbreviation: min/wk = minutes/week, MED = Median, METs = Metabolic Equivalents. * With excluding one missing datum from the median calculation. ** No Metabolic Equivalents value for sitting in IPQA-SF. a. denotes using short International Physical Activity Questionnaire (IPQA-SF).

## Data Availability

The identified datasets analyzed during the current study are available from the corresponding author on reasonable request.
